# Assessing physical symptoms, daily functioning, and well‐being in children with achondroplasia

**DOI:** 10.1002/ajmg.a.61903

**Published:** 2020-10-20

**Authors:** Kathryn M. Pfeiffer, Meryl Brod, Alden Smith, Jill Gianettoni, Dorthe Viuff, Sho Ota, R. Will Charlton

**Affiliations:** ^1^ Health Outcomes Research The Brod Group California USA; ^2^ Market Access Ascendis Pharma, Inc. Palo Alto California USA; ^3^ Clinical Operations, Ascendis Pharma, Inc. Palo Alto California USA; ^4^ Strategy & Project Management Ascendis Pharma A/S, Hellerup Denmark; ^5^ Clinical Development Ascendis Pharma, Inc. Palo Alto California USA

**Keywords:** achondroplasia, emotional well‐being, observer‐reported outcome measure, physical functioning, quality of life, social well‐being

## Abstract

This study's purpose was to provide qualitative evidence to support the development of two observer‐reported outcome measures assessing the physical symptoms/complications of achondroplasia in children and impacts on children's quality of life. Individual/focus group concept elicitation interviews were conducted with parents of children aged 2 to <12 years with achondroplasia and experts. Qualitative analysis of transcripts, based on an adapted grounded theory approach, informed item generation and measure development. Cognitive debriefing (CD) interviews were conducted with parents to confirm relevance and understanding. Thirty‐six parents participated in concept elicitation interviews. The analysis identified major physical symptoms/complications and impacts of achondroplasia, which informed the development of the Achondroplasia Child Experience Measures (ACEMs): ACEM—Symptom and ACEM—Impact. ACEM—Symptom was comprised of eight major symptoms/complications including pain (58%), ear infections/fluid in ear (56%), and low stamina/tiring easily (56%). ACEM—Impact consisted of 31 major impacts in the domains of daily functioning, emotional well‐being, social well‐being, and need for assistance/adaptive devices. Impacts on functioning included difficulty reaching objects/high places (89%) and toileting (67%). Emotional impacts included feeling different (53%) and feeling frustrated/annoyed (47%). Social impacts included difficulty participating in sports/physical play (86%) and being treated as younger than age (83%). Following CD interviews with 16 additional parents, validation‐ready ACEM measures were generated. The study improves our understanding of the experiences of children with achondroplasia and provides evidence supporting the content validity of the ACEMs. Validated ACEMs may be used to assess potential benefits of future treatments for comorbidities of achondroplasia.

## 1 INTRODUCTION

Achondroplasia is the most common form of dwarfism, affecting more than 250,000 people globally and with an estimated incidence of between one in 10,000 and one in 30,000 live births (Horton, Hall, & Hecht, 2007). Achondroplasia occurs as a result of a mutation in the *FGFR3* gene that affects bone and cartilage growth (Horton et al., 2007; Klag & Horton, 2016). Clinical features of achondroplasia typically include small stature, disproportional shortening of the legs and arms, macrocephaly with frontal bossing, midface hypoplasia, small chest size, abnormal curvature of the spine (kyphosis, lordosis), short fingers and trident‐shaped hands, joint hypermobility in hips and knees, and tibial bowing (Pauli, 2019).

The medical impacts and clinical complications of achondroplasia in children have been well‐studied (Hunter, Bankier, Rogers, Sillence, & Scott Jr., 1998; Pauli, 2019). Recurrent ear infections (otitis media), sleep apnea, hearing loss, teeth crowding/misalignment, and speech delay or articulation problems are common complications of achondroplasia in childhood (Hunter et al., 1998; Wright & Irving, 2012). Other possible complications in childhood include back, leg, and joint pain, respiratory issues, spinal stenosis resulting in neurological symptoms (e.g., tingling/numbness in extremities), hydrocephalus, and foramen magnum compression (Hunter et al., 1998; Wright & Irving, 2012). In adults with achondroplasia, complications include chronic back and leg pain, spinal stenosis, sleep apnea, and obesity, which contribute to increased mortality risk (Hunter et al., 1998; Pauli, 2019). Although there is no treatment for achondroplasia, guidelines for the medical management of achondroplasia and related complications have been established (Horton et al., 2007; Ireland et al., 2014; Pauli, 2019; Trotter, Hall, & American Academy of Pediatrics Committee on, 2005; Unger, Bonafe, & Gouze, 2017; Wright & Irving, 2012). New treatments aimed at normalizing bone and cartilage growth in children with achondroplasia are currently in clinical development (Breinholt et al., 2019; Savarirayan et al., 2019).

While the clinical complications and medical impacts of achondroplasia in children are well studied, there is limited research on broader impacts on children's lives, including daily functioning, emotional well‐being, and social well‐being (Bloemeke et al., 2019; Dogba, Rauch, Douglas, & Bedos, 2014; Gollust, Thompson, Gooding, & Biesecker, 2003; Sommer et al., 2017). Children with achondroplasia experience a lower general quality of life, on average, in terms of physical, emotional, social, and school functioning, compared to a reference population (Witt et al., 2019). Infants and young children with achondroplasia experience delays in some developmental milestones, including gross motor, fine motor, communication, and feeding milestones (Ireland et al., 2010; Ireland et al., 2012). At the age of 7 years, many children with achondroplasia continue to require parent/caregiver assistance with basic self‐care, including dressing/undressing, toileting, bathing, and grooming, and some children still require supervision in social settings (Ireland et al., 2011). Many require assistance or adaptive devices for activities of daily living such as self‐care and mobility (Ireland et al., 2014).

Currently, there are no patient‐reported outcome (PRO) or observer‐reported outcome (ObsRO) measures of the physical symptoms/complications of achondroplasia in children or the impacts of achondroplasia on children's functioning and well‐being that have been developed in accordance with the United States (US) Food and Drug Administration (FDA) guidelines for PRO measure development and best research practices (Brod, Tesler, & Christensen, 2009; Food and Drug Administration (FDA), 2009; Lasch et al., 2010; Patrick et al., 2011a). An ObsRO measure is an informant reported outcome measure and is recommended by the FDA to be used to measure treatment benefit or risk in medical product clinical trials when patients are not able to report outcomes themselves, as in the case of young children. An ObsRO measure is not a proxy measure requiring the informant (e.g., parent) to make interpretations or inferences about a child's subjective experience, but rather reflects informant reports of observable behavior in the child or observations of what the child has said (Matza et al., 2013).

The purpose of this study was to use rigorous and patient‐centered qualitative research methods to investigate the physical symptoms/complications of achondroplasia and the impacts of achondroplasia on daily functioning, emotional well‐being, and social well‐being in children ages 2 to <12 years. Further, these data were intended to inform the development, in accordance with FDA guidelines, of 2 ObsRO measures: (a) to assess the physical symptoms/complications of achondroplasia in children; and (b) to assess the impacts of achondroplasia on children's functioning and well‐being (Food and Drug Administration (FDA), 2009).

## 2 METHODS

### 2.1 Editorial policies and ethical considerations

The study was approved by an independent Institutional Review Board (IRB), Copernicus Group IRB, located in Research Triangle Park North Carolina, US (Protocol numbers TBG1‐18‐117 and 201905578), and was conducted in accordance with the 1964 Helsinki Declaration and its later amendments or comparable ethical standards. Informed consent was obtained from all parent participants prior to the telephone/focus group interviews.

### 2.2 Qualitative research study design and analysis

The qualitative research study design followed the FDA guidelines and best research practices for the development of PRO and ObsRO measures (Brod et al., 2009; Food and Drug Administration (FDA), 2009; Lasch et al., 2010; Patrick et al., 2011a). A targeted literature review and concept elicitation interviews with clinical/other experts in achondroplasia were conducted to provide relevant background information and clinical knowledge. Concept elicitation interviews were also conducted with parents of children aged 2 to <12 years with achondroplasia to obtain the patient perspective, which is the gold standard for ObsRO measure development.

A qualitative analysis of concept elicitation data was conducted based on an adapted grounded theory approach (Lasch et al., 2010). The qualitative analysis informed the development of the parent ObsRO measures and a preliminary theoretical model of the physical signs/symptoms and impacts of achondroplasia in children. Cognitive debriefing interviews for the ObsRO measures were then conducted with an additional sample of parents of children with achondroplasia to ensure that the measure items and instructions were clear, relevant, and inoffensive, that appropriate recall periods were used, and that response options and scales were understood easily and were appropriate for all items.

#### 2.2.1 Concept elicitation

Interviews with experts in achondroplasia were conducted to obtain relevant background information and clinical insights regarding achondroplasia in children and its impacts on children and parents. Expert telephone interviews were conducted individually with clinical experts in the US and Spain and a patient advocacy leader for achondroplasia in the US using a semi‐structured interview guide. To be eligible for participation in the interviews, clinical experts were required to have: (a) expertise in one or more field(s) relevant to achondroplasia, including pediatrics, clinical genetics, skeletal dysplasias, orthopedics, clinical psychology, and/or other relevant specialties; and (b) at least 5 years of experience treating children with achondroplasia in a clinical setting.

Based on the literature review and expert interviews, a semi‐structured interview guide was developed to elicit parents' observations related to their child's achondroplasia, including physical signs, symptoms, and complications, impacts on functioning and daily life, and impacts on emotional and social well‐being. Study inclusion criteria for the parents required participants to be: (a) adults aged 18 years or older; (b) able to read, write, and speak English (US) or Spanish (Spain); (c) the parent/guardian of a child (2 to <12 years of age) diagnosed with achondroplasia; and (d) actively involved in the child's care. Parents were ineligible for the study if they had a cognitive impairment or other medical condition that would affect their ability to participate in a telephone interview or focus group regarding their child with achondroplasia. These interviews were part of a larger study, which included interviews with parents of children <18 years of age with achondroplasia, as well as interviews with older children/adolescents aged 9 to <18 years with achondroplasia. Given the differences in the experiences of infants and older children/adolescents with achondroplasia, these data are analyzed separately, and results will be reported in separate manuscripts.

Several strategies were employed to recruit parent participants, including: (a) advocacy organizations for achondroplasia in the US and Spain assisted in disseminating the study information sheet to their membership through email and/or social media; (b) clinical expert interview participants were asked to share the study information sheet with parents who may be interested in participating; (c) a professional market research organization assisted with recruiting study participants through social media; and (d) a “snowball” sampling strategy, in which parent participants were asked to share the study information sheet with other parents they knew who may be interested in participating, was used. Recruiting targets were set for participants by country, child's age, and whether the parent also has achondroplasia to ensure that a broad range of parent experiences was captured through the interviews.

Before study participation, parents were required to verify their eligibility to participate in the study through a telephone screening interview. All participants received a modest honorarium for their participation.

Expert interviews were conducted by telephone, in English or Spanish based on expert preference, and lasted ~60 min. Individual concept elicitation interviews with parents were conducted by telephone and lasted ~60 min. One in‐person parent focus group was conducted in Spain and lasted approximately 2 hr. Due to logistical challenges, a parent focus group was not held in the US. Parent interviews were conducted in English (US) or Spanish (Spain) by experienced qualitative interviewers. All individual/focus group interviews were recorded and transcribed verbatim. Interviews conducted in Spanish were translated into English by a professional translation company.

Interview transcripts were analyzed for conceptual themes through an iterative process and based on an adapted grounded theory approach (Lasch et al., 2010). Qualitative data analysis was conducted using Dedoose©, a web‐based application for qualitative research (Dedoose Version 8.0.35, 2018). Based on the semi‐structured interview guides, a preliminary code list of concepts, including physical signs/symptoms and impacts, was developed. Interview transcripts were coded in chronological order by date conducted. Concepts that emerged during the coding process were added to the code scheme, and previously coded transcripts were then evaluated for the new concepts. Throughout the coding process, concepts/codes were organized into categories encompassing larger themes and sub‐themes.

To confirm the adequacy of the sample size to cover all relevant themes in the interviews, a thematic saturation analysis was conducted (Lasch et al., 2010). Thematic saturation was assessed for the 36 parent participants in the chronological order in which the interviews/focus group occurred. Parent reports of signs/symptoms and impacts reflect the number and percentage of parents who discussed each sign/symptom or impact during their interview. Therefore, there may be signs/symptoms or impacts experienced by their children that parents did not discuss during the interviews.

#### 2.2.2 Item generation

To generate items and measures, a two‐day, in‐person item generation meeting was held for each measure with the entire project team, including interviewers, the Scientific Director, and qualitative analysts. The team reviewed the qualitative data analysis report and revised or confirmed codes as needed. After the review, the team decided on criteria for identifying a physical symptom/complication or impact as major, and therefore included as a potential item in the measures, or minor. In line with FDA guidelines and best practices for ObsRO measure development, the team aimed to develop criteria for identifying symptoms/complications and impacts that were important and relevant to the experiences of children with achondroplasia of differing ages (including ages 2 to <5 years; ages 5 to <9 years; and ages 9 to <12 years). The agreed‐upon criteria for identifying symptoms/complications and impacts as major included:

1. Endorsement by at least 30% of parent participants in at least two of the three child age groups analyzed; or an endorsement by 25–29% of parent participants in at least two of the three age groups if conceptually important
2. Potentially responsive to a new treatment for children that would normalize bone and cartilage growth
3. Considered bothersome, limiting, or difficult
4. For physical symptoms/complications domain, must not be a characteristic of the condition
5. Impacts must be temporally proximal (rather than distal)

For symptoms/impacts that were not considered major, the criteria for identifying a symptom or impact as minor included:

1. Endorsement by at least 10% of participants in at least one of the three child age groups analyzed
2. Impacts must be temporally proximal (rather than distal)

To help ensure that the measures would be responsive to change without floor or ceiling effects, only major symptoms/complications and impacts were considered for inclusion as items in the measures.

Following the identification of major and minor symptoms/complications and impacts, a preliminary theoretical model and conceptual framework for the symptoms/complications and impacts of achondroplasia in children aged 2 to <12 years was developed. The purpose of the theoretical model was to illustrate the hypothesized relationships among the symptoms/complications and impacts of achondroplasia in children, as well as to identify potential factors which may modify physical symptoms/complications or impacts on children's daily functioning and well‐being.

Based on the qualitative analysis report, the preliminary theoretical model, and major symptoms/complications and impacts in the identified domains, two‐parent ObsRO measures for the symptoms and impacts of achondroplasia in children, collectively called the Achondroplasia Child Experience Measure (ACEM), were developed: (a) the ACEM—Symptom measures the physical symptoms and complications of achondroplasia in children aged 2 to <12 years; and (b) the ACEM—Impact measures the impacts of achondroplasia on the general functioning and well‐being of children aged 2 to <12 years. An item definition table was developed for each measure using parent participants' language to define the conceptual meaning of each instruction and item in the measure. A translatability assessment was then conducted to identify potential challenges to translating the measures into other languages and to suggest alternative wording to facilitate translation.

#### 2.2.3 Cognitive debriefing interviews

The purpose of the cognitive debriefing interviews was to ensure parent understanding and to confirm the relevance and comprehensiveness of the measures (Patrick et al., 2011b). The cognitive debriefing interviews aimed to confirm that the structure and format of the measures were appropriate, that all measure instructions, items, and response options were understood easily, and that recall periods were appropriate. The cognitive debriefing interviews also aimed to confirm that the content of the items and measures was relevant and that the measures did not omit any important symptoms/complications or impacts.

Individual cognitive debriefing interviews were conducted by telephone in the US with an independent sample of parents of children with achondroplasia aged 2 to <12 years. Parent participants in the cognitive debriefing interviews were required to meet the same eligibility criteria used for the concept elicitation interview participants and were recruited using the same multi‐pronged approach described above. All participants were emailed the two measures in advance and were asked to complete them 24–48 hr prior to their scheduled interview and to have the completed measures with them for reference during the interview.

The interviews were conducted based on a structured interview guide, which used a “think aloud” method and verbal probing to ask respondents questions about the measures (Patrick et al., 2011b). Parents were asked to explain their thought process when responding to each question to confirm that responses were based on their observations (e.g., what they saw or heard from their child or what they heard from others who know their child well). Only ObsRO measure items that parents could answer based on their observations would be included in the measures.

Cognitive debriefing interviews lasted ~90 min and were conducted in blocks of three participants each. Following the first block, the research team reviewed the findings and decided on any changes needed in the measures. This process was repeated with blocks of three parent participants until the participants in the block reached a consensus that the readability and relevance of the measures were acceptable and that no additional revisions to the measures were needed. Once the cognitive debriefing interviews were completed, validation‐ready versions of the measures were created.

## 3 RESULTS

### 3.1 Concept elicitation interviews

#### 3.1.1 Expert and parent sample descriptions

Seven experts in the US (*n* = 4) and Spain (*n* = 3) participated in concept elicitation interviews. One expert in the US was a leader in an achondroplasia advocacy organization, and six experts were clinical specialists with expertise in achondroplasia. Clinical experts had a range of different specialty backgrounds, including clinical/medical genetics (*n* = 3), pediatrics (*n* = 1), primary care (*n* = 1), skeletal dysplasias (*n* = 1), orthopedic surgery (*n* = 1), traumatology (*n* = 1), and clinical psychology (*n* = 1). Average years of experience in their clinical/medical specialty was 26 (range, 14–40 years). Mean years of experience treating patients with achondroplasia was 23 (range 5–40 years). Two clinical experts (33%) worked in an academic hospital setting, two experts (33%) worked in a public/other hospital, one expert (17%) worked in a clinic, and one expert (17%) was recently retired.

Thirty‐six parents of children with achondroplasia aged 2 to <12 years participated in individual telephone or focus group interviews. Average parent age was 41.5 years (range, 32–68). Thirty‐one parent participants were mothers (86%), and five were fathers (14%). Most participants were married (81%, *n* = 29), 8% were partnered (*n* = 3), 6% were divorced (*n* = 2), and 6% were single (*n* = 2). Parent racial/ethnic background was measured in the US only, and response categories were not mutually exclusive. White/Caucasian was the most frequently reported racial/ethnic group (92%, *n* = 23), followed by Black/African American (12%, *n* = 3), and Latino/Hispanic (4%, *n* = 1). Three parents (8%) had less than high school education, six parents (17%) had a high school degree/equivalent, 17 parents (47%) had a college degree, and 10 parents (28%) attended post‐graduate school.

In total, 31% of parent participants (*n* = 11) had children aged 2 to <5 years with achondroplasia, 36% of parents (*n* = 13) had children aged 5 to <9 years, and 33% of parents (*n* = 12) had children aged 9 to <12 years. Fifty‐three percent of parent participants (*n* = 19) had daughters and 47% (*n* = 17) had sons with achondroplasia. The most frequently reported racial/ethnic background of the children of US parent participants was White/Caucasian (80%, *n* = 20), followed by Black/African‐American (16%, *n* = 4), Latino/Hispanic (8%, *n* = 2), and Asian‐American (8%, *n* = 2). In total, 33% of parents (*n* = 12) described their child's health as “excellent,” 39% (*n* = 14) reported child's health as “very good,” 19% (*n* = 7) indicated child's health as “good,” and 8% (*n* = 3) reported child's health as “fair.”

#### 3.1.2 Expert concept elicitation interviews

Although experts discussed some variations in signs/symptoms and impacts related to child age, it was not possible to report signs/symptoms and impacts only relevant to children aged 2 to <12 years because experts tended to speak more generally about child age. Thus, expert reports reflect more general discussion of signs/symptoms and impacts relevant for children less than 18 years of age with achondroplasia.

Experts discussed many different physical signs and symptoms/complications experienced by children with achondroplasia less than 18 years of age. Clinical features of achondroplasia related to bone and cartilage growth, including short stature, disproportionate shortening of the arms and legs, macrocephaly, midfacial retrusion, small chest, kyphosis, hyperlordosis, limited elbow extension, short fingers and trident configuration of the hands, hypermobile hips and knees, bowing of the legs, and hypotonia (Pauli, 2019) are not discussed here as they are characteristic of the condition. The physical symptoms/complications of achondroplasia in children most frequently discussed by experts included, sleep apnea (71%), ear infections/fluid in ear (57%), hearing problems/loss (57%), overweight/obesity (57%), pain (e.g., in back, 43%), speech issues (43%), respiratory issues (not associated with sleep apnea, 43%), spinal stenosis/compression (43%), and foramen magnum stenosis/compression (43%).

Experts discussed many impacts on children's functioning and daily life. The most frequently mentioned impacts included difficulty reaching objects or high places (71%), difficulty walking long distances (71%), difficulty participating in class/schoolwork (71%), issues toileting self (57%), difficulty with prolonged sitting or sitting without support (57%), issues with bathing/washing/grooming self (43%), difficulty running (43%), difficulty being physically active (43%), and difficulty with tasks requiring fine motor skills (43%). Expert reports related to the use of adaptations/assistance from others included the use of adaptive devices outside of school (86%), having accommodations or other adaptations at school (e.g., more time to take tests; 86%), use of adaptive devices at school (57%), and needing assistance from others outside of school (43%).

Experts also reported on how achondroplasia affects children's emotional and social well‐being. The most often reported emotional impacts on children included feeling different (100%), confidence or self‐esteem issues (100%), difficulty accepting condition (71%), feeling alone/left out (57%), feeling that things are difficult/challenging (57%), feeling frustrated/annoyed (43%), feeling depressed/sad (43%), and having high confidence (43%). The most frequently mentioned impacts on children's social well‐being included difficulty participating in sports or physical play (100%), general social issues (e.g., “fitting in;” 100%), the experience of teasing/bullying (86%), difficulty participating in social activities (71%), being stigmatized by others (71%), making social connections/friendships in the achondroplasia/dwarfism community (71%), negative attention in public (e.g., staring; 57%), and social exclusion (57%).

#### 3.1.3 Parent concept elicitation interviews

In total, 175 concepts related to the physical signs, symptoms, and complications of achondroplasia in children (35 concepts) and the impacts of achondroplasia on children, parents, and families (140 concepts) were identified and coded in the parent individual and focus group interview transcripts (data not shown). The analysis of thematic saturation showed that after the sixth parent participant, 75% of concepts had been discussed. Following the 23rd participant, saturation was considered reached, with 96% of the concepts covered.

Parents discussed many physical signs, symptoms, and complications related to achondroplasia that they observed in their children. The most often mentioned physical symptoms/complications of achondroplasia, excluding clinical features of the condition (Pauli, 2019), are given in Table 1. The most frequently reported physical symptoms/complications were pain (e.g., in back, legs, joints; 58%), ear infections/fluid in ears (56%), low stamina/tiring easily (56%), hearing problems/loss (36%), balance issues/falls (36%), sleep apnea (33%), speech issues (e.g., delayed speech, difficulty with words; 33%), and trouble breathing while awake (excluding sleep apnea; 28%).

**TABLE 1:** Most frequently reported physical symptoms/complications of achondroplasia in children

	Parent reports by child age							
	2 to <5 years (*n* = 11)		5 to <9 years (*n* = 13)		9 to <12 years (*n* = 12)		Total (*n* = 36)	
*Reported physical symptoms/complications (n, %)*							
Pain	3	27%	11	85%	7	58%	21	58%
Ear infections/fluid in ear	9	82%	8	62%	3	25%	20	56%
Low stamina/tiring easily	6	55%	8	62%	6	50%	20	56%
Hearing problems/loss	3	27%	6	46%	4	33%	13	36%
Balance issues/falls	7	64%	4	31%	2	17%	13	36%
Sleep apnea	5	45%	2	15%	5	42%	12	33%
Speech issues	5	45%	5	38%	2	17%	12	33%
Trouble breathing while awake	3	27%	4	31%	3	25%	10	28%
Sweating/hot	3	27%	3	23%	2	17%	8	22%

*Note:* Clinical features of achondroplasia (e.g., short stature, disproportionate shortening of arms/legs, macrocephaly, etc.) discussed by parents are not included in this table.

Parents also reported impacts on children's functioning and school participation. The most often discussed impacts of achondroplasia on children's daily functioning and school participation are shown in Table 2. The most frequently mentioned impacts on functioning and daily life included difficulty reaching objects/high places (89%), issues toileting self (67%), difficulty bathing/showering, washing, and/or grooming (58%), difficulty running (56%), difficulty walking long distances (50%), difficulty being physically active (47%), issues with dressing/undressing (47%), difficulty climbing stairs/steps (42%), difficulty sitting for long periods or sitting without support (42%), and difficulty performing tasks requiring fine motor skills (e.g., grasping objects, writing; 39%). Frequently reported impacts on school participation related to achondroplasia included missed school days or time (58%), limited or modified participation in physical education/gym class (50%), and difficulty participating in class/schoolwork (36%).

**TABLE 2:** Most frequently reported impacts on children's functioning and school participation

	Parent reports by child age							
	2 to <5 years (*n* = 11)		5 to <9 years (*n* = 13)		9 to <12 years (*n* = 12)		Total (*n* = 36)	
*Impacts on functioning/daily life (n, %)*							
Reaching objects/high places	9	82%	12	92%	11	92%	32	89%
Toileting	8	73%	10	77%	6	50%	24	67%
Bathing/washing/grooming	5	45%	8	62%	8	67%	21	58%
Running	5	45%	8	62%	7	58%	20	56%
Walking	6	55%	7	54%	5	42%	18	50%
Being physically active	5	45%	6	46%	6	50%	17	47%
Dressing/undressing	8	73%	6	46%	3	25%	17	47%
Difficulty with stairs or steps	7	64%	4	31%	4	33%	15	42%
Prolonged sitting or sitting without support	4	36%	6	46%	5	42%	15	42%
Tasks requiring fine motor skills	5	45%	4	31%	5	42%	14	39%
Ability to travel	5	45%	5	38%	3	25%	13	36%
Communication	2	18%	5	38%	2	17%	9	25%
*Impacts on school participation (n, %)*								
Missed school days/time	2	18%	10	77%	9	75%	21	58%
Participation in physical education	1	9%	7	54%	10	83%	18	50%
Participation in class/schoolwork	3	27%	5	38%	5	42%	13	36%
Participation in school activities or field trips	2	18%	4	31%	2	17%	8	22%
Difficulty getting from place to place at school	0	0%	5	38%	3	25%	8	22%
*Adaptations/assistance outside of school (n, %)*								
Use of adaptive devices	9	82%	13	100%	12	100%	34	94%
Assistance from others	9	82%	12	92%	10	83%	31	86%
*Adaptations/assistance at school (n, %)*								
Use adaptive devices at school	5	45%	10	77%	10	83%	25	69%
Assistance from others at school	4	36%	6	46%	4	33%	14	39%
Other accommodations/adaptations (e.g., more time to take tests)	3	27%	10	77%	9	75%	22	61%

Parents also discussed children's need for adaptations or assistance in daily life. The most often mentioned adaptations used outside of school included adaptive devices (94%) and assistance from others for daily tasks (86%). Reported adaptations in school included the use of adaptive devices at school (69%), having other accommodations/adaptations at school (e.g., more time to take tests or to get from place to place; 61%), and needing assistance from others at school (39%).

In addition to functioning and daily life, parents also reported impacts on children's emotional and social well‐being related to achondroplasia (Table 3). The most often mentioned emotional impacts included feeling different from others (53%), feeling frustrated/annoyed (47%), feeling depressed/sad (39%), feeling angry/mad (33%), feeling embarrassed/self‐conscious (33%), general upset (31%), difficulty accepting condition (28%), expecting special treatment or using achondroplasia as an excuse (25%), feeling worried/scared (22%), and feeling bad/hurt (22%). The most frequently reported impacts on children's social well‐being were difficulty participating in sports/physical play (86%), being treated as younger than age (83%), negative attention in public (e.g., staring, pointing; 64%), the experience of teasing/bullying (64%), difficulty participating in social activities (e.g., birthday parties, playdates; 64%), difficulty keeping up with other children their age physically (58%), being stigmatized by others (56%), needing to explain achondroplasia to others (53%), and connecting with a community of people with achondroplasia/dwarfism (42%).

**TABLE 3:** Most frequently reported impacts on children's emotional and social well‐being

	Parent reports by child age							
	2 to <5 years (*n* = 11)		5 to <9 years (*n* = 13)		9 to <12 years (*n* = 12)		Total (*n* = 36)	
*Emotional impacts/issues (n, %)*							
Feeling different	2	18%	8	62%	9	75%	19	53%
Frustrated/annoyed	5	45%	7	54%	5	42%	17	47%
Depressed/sad	1	9%	6	46%	7	58%	14	39%
Angry/mad	4	36%	5	38%	3	25%	12	33%
Embarrassed/self‐conscious	1	9%	6	46%	5	42%	12	33%
General upset	3	27%	3	23%	5	42%	11	31%
Difficulty accepting condition	1	9%	6	46%	3	25%	10	28%
Special treatment/excuse	2	18%	3	23%	4	33%	9	25%
Worried/scared	0	0%	4	31%	4	33%	8	22%
Bad/hurt	0	0%	2	15%	6	50%	8	22%
*Social impacts/issues (n, %)*								
Participation in sports or physical play	6	55%	13	100%	12	100%	31	86%
Treated as younger than age	8	73%	11	85%	11	92%	30	83%
Negative attention in public	8	73%	7	54%	8	67%	23	64%
Teasing/bullying	2	18%	11	85%	10	83%	23	64%
Participation in social activities	6	55%	11	85%	6	50%	23	64%
Keeping up with other children their age	6	55%	8	62%	7	58%	21	58%
Being stigmatized	4	36%	7	54%	9	75%	20	56%
Explaining ACH to others	1	9%	8	62%	10	83%	19	53%
ACH/dwarfism community	3	27%	5	38%	7	58%	15	42%
Unwanted touching/lifting	3	27%	7	54%	3	25%	13	36%
Being popular/well‐known	1	9%	7	54%	5	42%	13	36%
Peers treat differently	1	9%	3	23%	4	33%	8	22%

Abbreviation: ACH, achondroplasia.

Results were generally similar among parents with and without achondroplasia, although endorsement rates were lower for some of the symptoms/complications and impacts reported by parents with achondroplasia compared to parents without achondroplasia (data not shown). Given the small subsample sizes, statistical significance tests of differences were not conducted.

#### 3.1.4 Preliminary theoretical model

**FIGURE 1:**
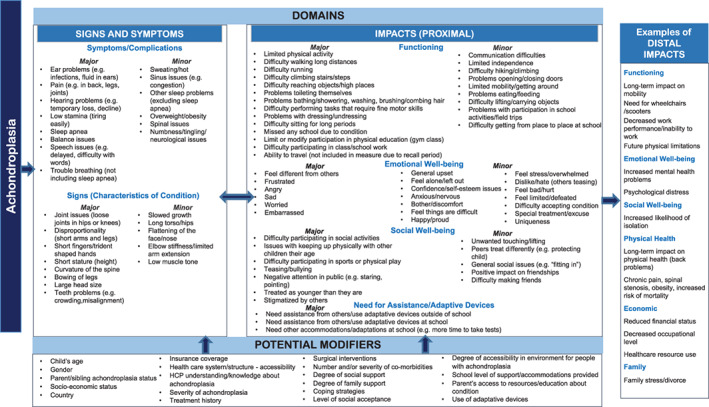
Preliminary theoretical model for symptoms, signs, and impacts of achondroplasia (children ages 2 to <12 years) [Color figure can be viewed at wileyonlinelibrary.com]

The qualitative analysis of concept elicitation interviews informed the development of the preliminary theoretical model of the symptoms/complications and impacts of achondroplasia on children's functioning and well‐being (Figure 1). The preliminary model outlines the hypothesized relationships among physical symptoms/complications and impacts of achondroplasia in children aged 2 to <12 years. Additionally, the model identifies major and minor symptoms/complications and impacts and distinguishes between temporally proximal and distal impacts. Further, the model specifies potential modifiers of symptoms/complications or impacts of achondroplasia in children, such as child's age, degree of family support, coping strategies, and treatment history.

### 3.2 Item generation

Based on the criteria for identifying major symptoms and impacts described above, drafts of the preliminary ACEM—Symptom and ACEM—Impact measures were generated. Following the translatability assessment report, minor edits to the wording in some items were made to avoid potential translation issues.

#### 3.2.1 Parent cognitive debriefing interviews

In total, 16 parents in the US participated in the cognitive debriefing telephone interviews. All parent cognitive debriefing interview participants were mothers (100%, *n* = 16), and the average parent age was 39.1 years (range, 31–55). Most parents (88%, *n* = 14) were married, one parent was single (6%), and one parent did not report marital status (6%). Fourteen parents (88%) indicated White/Caucasian racial/ethnic background, and one each reported Asian‐American (6%) and Latino/Hispanic (6%). For the highest completed education, two parents reported high school degree/equivalent (13%), one parent reported vocational/technical school (6%), nine parents reported college degree (56%), and four parents reported post‐graduate education (25%). In total, 44% of parents (*n* = 7) worked full‐time, 25% of parents (*n* = 4) worked part‐time, and 31% of parents (*n* = 5) were not currently employed. For household income, 6% reported household income between $40,001 and 60,000 (*n* = 1), 6% had household income from $60,001 to 80,000 (*n* = 1), 38% reported household income from $80,001 to 100,000 (*n* = 6), 38% reported household income greater than $100,000 (*n* = 6), and 13% (*n* = 2) declined to report. Two of the parent cognitive debriefing interview participants reported having achondroplasia (13%).

The age of the participants' children varied, with 25% of children (*n* = 4) aged 2 to <5 years, 38% of children (*n* = 6) aged 5 to <9 years, and 38% of children (*n* = 6) aged 9 to <12 years. Twelve children (75%) were female, and four (25%) were male. Most children had a White/Caucasian racial/ethnic background (69%, *n* = 11), followed by Asian‐American (25%, *n* = 4), and Latino/Hispanic (6%, *n* = 1). Most parents reported a child's health status as “excellent” (50%, *n* = 8) or “very good” (44%, *n* = 7), and one parent reported “fair” (6%).

Five blocks of parents were required to revise the measures and items to improve readability and relevance. A sixth, confirmatory block was conducted with three parents (two block 1 participants and 1 new participant) of preschool‐aged children to affirm the readability and relevance of questions related to school in this population.

#### 3.2.2 Preliminary measures

**FIGURE 2:**
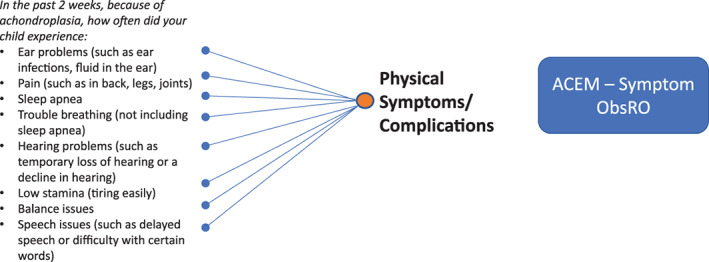
Preliminary conceptual framework of the Achondroplasia Child Experience Measure—Symptom (ACEM—Symptom) [Color figure can be viewed at wileyonlinelibrary.com]

**FIGURE 3:**
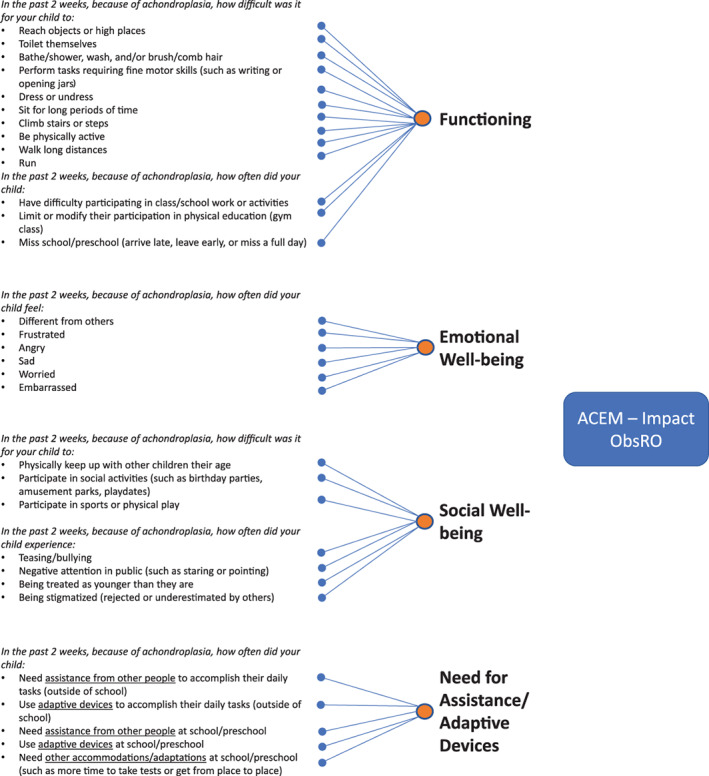
Preliminary conceptual framework of the Achondroplasia Child Experience Measure—Impact (ACEM—Impact) [Color figure can be viewed at wileyonlinelibrary.com]

The cognitive debriefing process culminated in validation‐ready versions of the ACEM—Symptom and ACEM—Impact parent ObsRO measures. The ACEM—Symptom ObsRO reflects one domain, Physical Symptoms/Complications of achondroplasia, and is composed of eight items using one question stem and corresponding response options. The preliminary conceptual framework of the ACEM—Symptom ObsRO is shown in Figure 2. The ACEM—Impact ObsRO reflects four domains: Functioning, Emotional Well‐being, Social Well‐being, and Need for Assistance/Adaptive Devices. This measure contains 31 items and uses seven different stems with appropriate response options. The preliminary conceptual framework for the ACEM—Impact ObsRO is given in Figure 3. Both measures are intended to be completed by parents or caregivers of children aged 2 to <12 years with achondroplasia who are involved in the daily care of their children.

All items in the ACEM measures included a five‐point, Likert‐type response scale, which allowed for meaningful distinctions among response options and minimal cognitive burden for respondents (Dillman, Smyth, & Christian, 2014). To reduce the risk of recall bias, the recall period for all items in the ACEM measures was set at 2 weeks. Items measuring difficulty had response scales ranging from “Not at all difficult” to “Extremely difficult.” For the stem/items measuring the amount of time, the response scale ranged from “None” to “A great deal.” Items measuring “how often” had response scales ranging from “Never” to “Very often/always.” In the ACEM—Symptom ObsRO, “percent of time” was indicated in parentheses for the response option scale measuring how often children experienced physical symptoms to make it easier for parents to provide accurate and consistent responses. Thus, for this stem and its corresponding items, response options ranged from “Rarely (1–25% of the time)” to “Very often/always (76–100% of the time).” An additional response option for “Don't know” was added for all items in the ObsRO measures to allow parents to indicate when they did not have enough information based on their observations to respond. For the stem and items measuring difficulty in child functioning/daily life in the ACEM—Impact measure, a response option of “My child CAN NOT DO without assistance/device” was added so that parents could indicate that their child always needed assistance or an adaptive device for a given task/activity.

## 4 DISCUSSION

Results suggest that achondroplasia affects children's lives in multiple ways, including impacts on children's day‐to‐day functioning and school participation, impacts on children's emotional and social well‐being, and the need for assistance, adaptive devices, and accommodations. The study is consistent with research on the physical signs/symptoms and medical impacts of achondroplasia in children (Horton et al., 2007; Klag & Horton, 2016; Pauli, 2019). Parents observed a wide range of physical signs, symptoms, and complications of achondroplasia in their children that varied somewhat by the child's age. The findings also add to previous studies of the impacts of achondroplasia on children's health, functioning and well‐being (Ireland et al., 2010; Ireland et al., 2011; Ireland et al., 2012; Witt et al., 2019).

In addition, the study provides qualitative evidence to support the content validity of the ACEM—Symptom and ACEM—Impact measures. These are the first achondroplasia‐specific ObsRO measures of physical symptoms/complications and impacts on quality of life in children that were developed in accordance with FDA guidelines and best practices for measure development (Brod et al., 2009; Food and Drug Administration (FDA), 2009; Lasch et al., 2010; Patrick et al., 2011a). The Achondroplasia Personal Life Experience Scale (APLES) measures the impacts of achondroplasia in children, but the authors acknowledge that the patient perspective was not fully considered in the development of APLES, which may limit the content validity to some degree (Bloemeke, Sommer, Witt, Dabs, et al., 2019; Sommer et al., 2017). Indeed, cognitive debriefing interviews conducted with APLES identified several important issues for children with achondroplasia that were not covered, including participation in a physical education class, visiting a playground, riding a bike, and help with toileting (Bloemeke, Sommer, Witt, Dabs, et al., 2019). The APLES also requires further psychometric validation and was developed for children starting at age 4, so would not be appropriate for children aged 2 to <4 years (Bloemeke, Sommer, Witt, Bullinger, et al., 2019; Bloemeke, Sommer, Witt, Dabs, et al., 2019). Other proposed measures for assessing the quality of life in children with achondroplasia (e.g., PedsQL, QoLISSY) are generic and therefore less likely to be as relevant and responsive as a condition‐specific measure (Bloemeke, Sommer, Witt, Bullinger, et al., 2019).

Additionally, the results reinforce the importance of considering the parent perspective, in addition to clinical expertise, in developing ObsRO measures for achondroplasia in children. Parent and expert reports of physical symptoms/complications and impacts sometimes differed greatly (though tests of statistical significance were not conducted). For instance, no expert mentioned children missing days or time at school due to achondroplasia, compared with 58% of parents. Moreover, all experts reported confidence/self‐esteem issues in children with achondroplasia compared with only 14% of parents. These differences suggest that parents and experts have varying, but equally important, insights into the experiences of children with achondroplasia in their care.

As new treatments for achondroplasia are being developed, it is critical for clinicians and researchers to assess the impacts of achondroplasia on children's physical symptoms/complications, daily functioning, and general well‐being that could potentially improve with future treatments. A PRO or ObsRO measure should assess symptoms and impacts that are relevant and important to patients (Food and Drug Administration (FDA), 2009). The ACEM—Symptom and ACEM—Impact measures will allow both researchers and clinicians to identify potential impacts of future treatments for achondroplasia, beyond clinical growth indicators such as height or annualized height velocity, that are meaningful to children and parents.

Due to the relatively broad child age range (2 to <12 years), a small number of relevant impacts for children in one age group may not be included as items in the ACEM. For instance, 46% of parents of children aged 5 to <9 years reported that children had difficulty accepting the condition, but this issue was less frequently reported by parents of younger and older children (Table 3). Some of these specific impacts, however, were included as higher‐order concepts. For example, although the ACEM—Impact does not have a specific item for general mobility or “getting around,” which was discussed by several parents of younger children, this concept would be covered in other ACEM—Impact items (e.g., difficulty walking long distances, running, and being physically active). Conversely, items that were included in the ACEM measures may be less relevant to children in one child age group compared to children in other age groups. For example, feeling different and feeling sad were reported by relatively few parents of children aged 2 to <5 years, but were much more frequently mentioned by parents of older children. The relatively broad child age range for the measures was designed so that ACEM could be used to characterize the course of outcomes over time. The broad age range would also allow ACEM to be a useful assessment tool in clinical trials of future treatments for achondroplasia in children that modulate bone and cartilage growth, which would likely require many years of treatment to realize the full benefit. Nevertheless, all symptoms/complications included in the ACEM—Symptom and all but one of the impacts included in the ACEM—Impact (feeling worried) were reported by parents in all three of the child age groups analyzed.

The findings also have important implications for the clinical management of achondroplasia in children (Horton et al., 2007; Ireland et al., 2014; Pauli, 2019; Trotter et al., 2005; Unger et al., 2017; Wright & Irving, 2012). Recognizing the broad spectrum of impacts on children's physical symptoms/complications, daily functioning and well‐being may improve communication among physicians, children with achondroplasia, and parents. Additionally, a better understanding of the challenges that children with achondroplasia face will better equip physicians and parents to provide children with support, including identifying and addressing challenges to daily activities and self‐care, helping children cope with emotional difficulties such as feeling different, addressing social issues such as staring and teasing/bullying, removing barriers to participation in social activities and sports/physical play, and advocating for children in school and other settings.

Several limitations should be acknowledged in interpreting the study results and measure development. The ACEM ObsRO measures rely on parent observations of child symptoms/complications and impacts associated with achondroplasia, which may differ from the perspective of the child. Research has shown that for chronic pediatric diseases, parents report lower child quality of life in parent proxy measures compared to child self‐reports (Upton, Lawford, & Eiser, 2008). The ACEMs are ObsRO measures, rather than proxy measures, and are thus designed to elicit parent observations of their children rather than parent reports on what they think their children experience. Given the potential for underestimating health‐related child quality of life in parent proxy reports, future measures of the impacts of achondroplasia on children's quality of life should be ObsRO measures, rather than proxy measures, when child self‐report is not possible.

Additionally, the qualitative study was based on a nonrandom sample, which may not be representative of all parents of children with achondroplasia. Despite challenges to study recruitment for rare conditions, the sample is diverse in terms of important demographic and background characteristics, and whether the parent also has achondroplasia. The analysis of thematic saturation suggests that the sample size was adequate to cover the broad range of experiences found in the study population, which is the aim of qualitative research studies.

Since the concept elicitation interviews were conducted in the US and Spain, the findings may not be generalizable to other countries with different cultures and/or healthcare systems. Statistical significance tests of sub‐group differences (e.g., among parents in different child age groups) in reported symptoms and impacts were not conducted due to small subsample sizes. Thus, reported differences between child age groups may not reflect actual differences in the population, and results should be interpreted with caution. Future research is warranted to investigate potential sub‐group or cross‐country differences further. As the ACEM measures assess the physical symptoms/complications and impacts associated with achondroplasia in children, future research should explore the development of PRO measures for older children/adolescents with achondroplasia to assess symptoms or impacts on quality of life relevant to this age group.

This study has shown broad impacts of achondroplasia on children aged 2 to <12 years, including experience of physical symptoms/complications and impacts on children's daily functioning and emotional and social well‐being. The identification of major physical symptoms and complications of achondroplasia and major impacts on functioning and well‐being in children provides evidence of the content validity of the ACEM ObsRO measures. Once validated, the ACEM will allow researchers and clinicians to assess the impacts of future treatments for achondroplasia on children's physical symptoms/complications and quality of life. The preliminary theoretical model developed for this study may also be a useful tool for informing research and clinical practice.

## CONFLICT OF INTEREST

K. M. Pfeiffer and M. Brod are consultants to the pharmaceutical industry including Ascendis Pharma. A. Smith and R. W. Charlton are employees of Ascendis Pharma, Inc. D. Viuff is an employee of Ascendis Pharma, A/S. J. Gianettoni and S. Ota were employees of Ascendis Pharma, Inc. when the research was conducted.

## AUTHOR CONTRIBUTIONS

All authors participated in the conception and design of the study. Kathryn M. Pfeiffer and Meryl Brod were involved in data collection, data analysis, interpretation of results, and drafting the manuscript. Alden Smith, Jill Gianettoni, Dorthe Viuff, Sho Ota, and R. Will Charlton contributed to critical manuscript revisions and intellectual content. All authors have given their approval of the final version of the manuscript and take responsibility for the manuscript's content and accuracy.

## Data Availability

Data Availability Statement: The data for the research presented in the publication may be available on a case by case basis for reasonable requests from the corresponding author.
